# Co-designing a structured referral template to enhance dementia diagnosis: a modified e-Delphi study

**DOI:** 10.1093/ageing/afag008

**Published:** 2026-02-02

**Authors:** Mary Cronin, Aisling A Jennings, Una Caufield, Isabelle Coonan, Nicola Cornally, Bart Daly, Lucinda Dockeray, Irene Hartigan, Brian Lawlor, Geraldine McCarthy, Aoife M Ni Chorcorain, Sean O’Dowd, Janice Nolan-Palmer, Marieke Perry, Diarmuid Quinlan, Suzanne Timmons, Tony Foley

**Affiliations:** Department of General Practice, School of Medicine, College of Medicine and Health, University College Cork, Cork, Ireland; Department of General Practice, School of Medicine, College of Medicine and Health, University College Cork, Cork, Ireland; Dementia Research Advisory Team, Alzheimer Society of Ireland, Dublin, Ireland; Department of General Practice, School of Medicine, College of Medicine and Health, University College Cork, Cork, Ireland; School of Nursing and Midwifery, University College Cork, Cork, Ireland; Integrated Care Programme of Older Persons (ICPOP), HSE South West and Cork University Hospital, Cork, Ireland; Irish College of General Practitioners, Dublin, Ireland; School of Nursing and Midwifery, University College Cork, Cork, Ireland; Trinity College Dublin School of Medicine - Trinity Institute of Neurosciences, Dublin, Leinster, Ireland; Sligo Medical Academy, University of Galway, Galway, Ireland; Old Age Psychiatry Department, Carlow Kilkenny Mental Health Services, Kilkenny, Ireland; National Dementia Services, Clonminch, Tullamore, Co Offaly, Ireland; PPI Contributor, Dublin, Ireland; Department of Primary and Community Care, Radboud University Medical Center, Nijmegen, Netherlands; Irish College of General Practitioners, Dublin, Ireland; Centre of Gerontology & Rehabilitation,School of Medicine, University College Cork, Cork, Ireland; Department of General Practice, School of Medicine, College of Medicine and Health, University College Cork, Cork, Ireland

**Keywords:** dementia, diagnosis, referral, co-design, primary care, older people

## Abstract

**Background:**

Dementia care is a health and social care priority, with rising prevalence driven by ageing populations worldwide. Timely and accurate diagnosis improves quality of life, enables access to support and is becoming even more critical due to the emergence of disease-modifying therapies for Alzheimer’s disease. Complex referral pathways can contribute to diagnostic delays and under-diagnosis. A structured, evidence-based referral template could enhance diagnostic efficiency and care quality.

**Methods:**

This study was conducted in two phases. First, a two-round e-Delphi survey was used to achieve consensus on items for inclusion in a dementia referral template. In the second phase, a modified Nominal Group Technique was employed with a multidisciplinary panel and Public and Patient Involvement (PPI) contributors to discuss, refine and prioritise items, ensuring clinical relevance and practical applicability.

**Results:**

The consensus process refined and prioritised 76 potential referral items into a final set of 11 essential components. The resulting concise template balances clinical relevance with usability, potentially supporting more efficient referral and triage. Items achieving the highest consensus included cognitive screening scores, rapid deterioration, problems with daily activities and patient safety concerns.

**Conclusion:**

The findings demonstrate the value of structured consensus methods in developing a practical, evidence-based referral template, tailored to optimise dementia diagnostic pathways. This is particularly important in the current evolving therapeutic landscape, to ensure that people with suspected dementia receive timely diagnosis and access to appropriate care and treatment options.

## Key Points

- A mixed-methods design using an e-Delphi and m-NGT established expert consensus on referral items.
- Co-design by clinicians and patient representatives ensured the template’s clinical relevance and utility.
- The consensus process distilled 76 potential items to a final, prioritised list of 11 for effective triage.
- The template balances the need for critical clinical data with the demands of primary care workload.
- This tool aims to standardise referrals and streamline the diagnostic pathway to reduce delays in care.

## Introduction

Dementia care is one of the most pressing health and social care challenges of the 21st century [1]. The prevalence of dementia is steadily rising as a direct consequence of ageing populations worldwide [2]. This demographic shift means that the impact on individuals, families and healthcare systems continues to grow, leading to increased economic and social burden [3]. Timely and accurate diagnosis is crucial in optimising the quality of life for individuals living with dementia, as early intervention allows for better symptom management and access to support services [4]. Furthermore, early identification enables the proactive management of modifiable risk factors, including cerebrovascular risks, which may help in reducing the rate of cognitive decline [5]. Beyond these established benefits, the introduction of disease-modifying therapies (DMTs) for Alzheimer’s disease (AD), the most common subtype of dementia, adds a compelling argument for the early identification of symptoms and accurate diagnosis [6]. Despite the well-established benefits of timely diagnosis, the average time to confirmation exceeds 3.5 years [7, 8], meaning that delayed detection and underdiagnosis remain significant challenges. Furthermore, many people experiencing cognitive decline do not receive a diagnosis until the condition has progressed to more advanced stages, potentially limiting the effectiveness of available treatments and interventions [9].

In most healthcare systems, the diagnostic pathway for suspected dementia typically begins in general practice, where general practitioners (GPs) serve as the first point of contact for patients and their families. GPs play a crucial role in early identification and assessment, before referral to specialist memory clinics for confirmation of the diagnosis and subtyping [10]. However, the referral process from primary to secondary care is often hindered by a lack of a standardised referral system, and a corresponding lack of consensus on what essential data should be included. This lack of clarity may lead to diagnostic delays, misdiagnosis, inefficient triage and missed opportunities for early intervention [11]. Ensuring that referrals contain the relevant information for triage and specialist assessment is vital, while minimising administrative burden and workload in primary care [11].

This study directly addresses this issue by developing a structured, bespoke referral template for referring people with suspected cognitive impairment from primary to secondary care.

The primary aim was to identify the essential items for a standardised referral template, as determined by a consensus-building process. Moreover, the successful development and implementation of a structured referral template has the potential to improve the efficiency of diagnostic pathways, reduce diagnostic delays and ultimately enhance the quality of care for people with dementia.

## Methods

The study was conducted in two interlinked phases. A two-round e-Delphi study was conducted to achieve consensus on the items to be included in a referral form, and this was followed by a modified Nominal Group Technique (m-NGT) to facilitate the co-design of the referral template. The sequential nature of the Delphi and m-NGT allowed for initial broad input and refinement, followed by focused discussion and final prioritisation [12]. The multidisciplinary research team included general practitioners, nurses, neurologists, geriatricians, psychiatrists and public and patient involvement (PPI) representatives, all key stakeholders in the dementia diagnostic pathway [13]. The inclusion of PPI contributors, both of whom were caregivers of people living with dementia, ensured that the perspectives of patients and caregivers were represented. To ensure the developed template was clinically relevant and practically applicable, the research team actively participated by reviewing survey questionnaires between e-Delphi rounds. They also assessed the final results to guide item categorisation and inclusion. Ethical approval was granted by the Social Research Ethics Committee at University College Cork (Ref:2024–119). This work was supported by an applied partnership award from the Health Research Board [APA-2022-027].

## Modified e-Delphi

### Survey design

A comprehensive scoping exercise was undertaken to identify potential items for inclusion in a referral form for patients with suspected cognitive impairment or dementia being referred to secondary care. Peer-reviewed databases, CINAHL and Medline, were searched using variations of the terms ‘*dementia,’* ‘*referral,’* and ‘*primary care.’* Additionally, searches were conducted on Google and Google Scholar, reviewing the first 200 citations. This process identified 15 existing referral templates from which items were extracted and reviewed by the research team to develop the first-round survey. The survey, comprising 76 items across 13 categories, was further reviewed by two PPI contributors and was piloted with five professionals from diverse clinical fields, including general practice, gerontology and physiotherapy. Minor adjustments were made, following this process, to refine clarity and usability.

### Participant recruitment and administering the survey

Clinicians with over 10 years of experience in dementia care were recruited to the e-Delphi panel via the research team’s professional networks. Invitation emails, containing a link to the first round of the e-Delphi survey, were distributed by research team members to their networks. The first-round survey remained open for 3 weeks, with follow-up reminder emails sent after one week to non-responders.

In each Delphi round, participants rated the importance of each proposed item using a 9-point Likert scale, with 1 indicating limited importance and 9 indicating critical importance. A free-text option allowed participants to provide comments and suggest additional items for consideration in the referral form. Four weeks after the completion of round one, the second-round survey was distributed, again followed by a reminder.

### Analysis

Responses were imported to SPSS for analysis. Following established Delphi methodology, a pre-defined consensus threshold was applied [14]. Specifically, items rated critically important by ≥80% of the panel (scoring 8–9 on Likert scale) in Round 1 were retained. Items rated as critically important by 60%–80% of the panel were re-presented in Round 2 [15]. To facilitate reconsideration of their scores, participants received anonymised feedback on the group’s median scores and their own previous scores for these items. Items deemed less relevant (<60%) were excluded. Suggestions provided through the free-text option were systematically reviewed and incorporated into the Round 2 survey if proposed by two or more panel members. To ensure that the final template would integrate smoothly with Ireland’s existing digital healthcare systems, the 39 items identified through the Delphi process were cross-referenced against the generic referral template used by HealthLink. HealthLink serves as the national platform that standardises the majority of electronic referrals from general practice to secondary care services in Ireland. In addition, any items that were similar were merged, resulting in a final, consolidated list for the m-NGT process.

### Modified nominal group technique

Following the e-Delphi rounds, an m-NGT was employed. The traditional NGT typically involves four key stages: (i) silent generation of ideas in response to a question, (ii) round-robin feedback where each participant shares one idea at a time, (iii) group discussion to clarify and consolidate items and (iv) individual ranking or voting to prioritise the most essential items [16]. In our study, the idea-generation stage had already been addressed through the preceding Delphi process. Accordingly, the traditional NGT was adapted to focus on ranking, round-robin contributions and facilitated discussion of the Delphi-derived items, followed by structured voting. This ensured that the final decision reflected a variety of expert opinions, while minimising the influence of dominant personalities [17]. The m-NGT session was conducted remotely via Microsoft Teams. The m-NGT panel was composed of the study’s research team, a group of multidisciplinary experts with considerable experience in dementia care pathways. To ensure a productive and focused discussion, research team members were sent a preparatory ranking task a week in advance of the meeting, asking them to rank their top 10 items from the items remaining after the Delphi rounds [18]. The meeting combined traditional round-robin discussion and clarification into a facilitated, focused discussion led by TF and guided by three questions: whether items were acceptable in practice, clinically relevant, and valuable for triage in memory clinics [18]. In the final voting stage of the m-NGT, members voted ‘yes,’ ‘no,’ or ‘undecided’ on whether an item was essential for inclusion [12]. This approach prioritised items deemed essential over those considered useful, but not critical [19]. This deliberate strategy aimed to achieve a concise, highly prioritised list of items, effectively minimising administrative burden for referring GPs, whilst ensuring relevant clinical information for secondary care professionals.

## Results

The two-round e-Delphi study was conducted over a three-month period. Eighty-five clinicians participated in Round 1, with 72% (61 participants) continuing into Round 2. The panel comprised healthcare professionals from both primary and secondary care settings, including GPs, geriatricians, neurologists, psychiatrists, nurses and allied health professionals. Table 1 provides demographic details of the participants, highlighting a diverse range of professional backgrounds and considerable experience in dementia care.

**Table 1:** Participant characteristics, round 1 and round 2

	Round 1	Round 2
**Professional role**	**N**	**N**
General practitioner	29	22
Geriatrician	14	9
Neurologist	8	6
Psychiatrist	10	6
Physiotherapist	4	3
Occupational therapist	3	3
Nurse	15	11
Other	2	1
**Years of experience in dementia care**		
10–20	59	44
21–30	18	11
31–40	5	4
> 40	3	2

**Figure 1:**
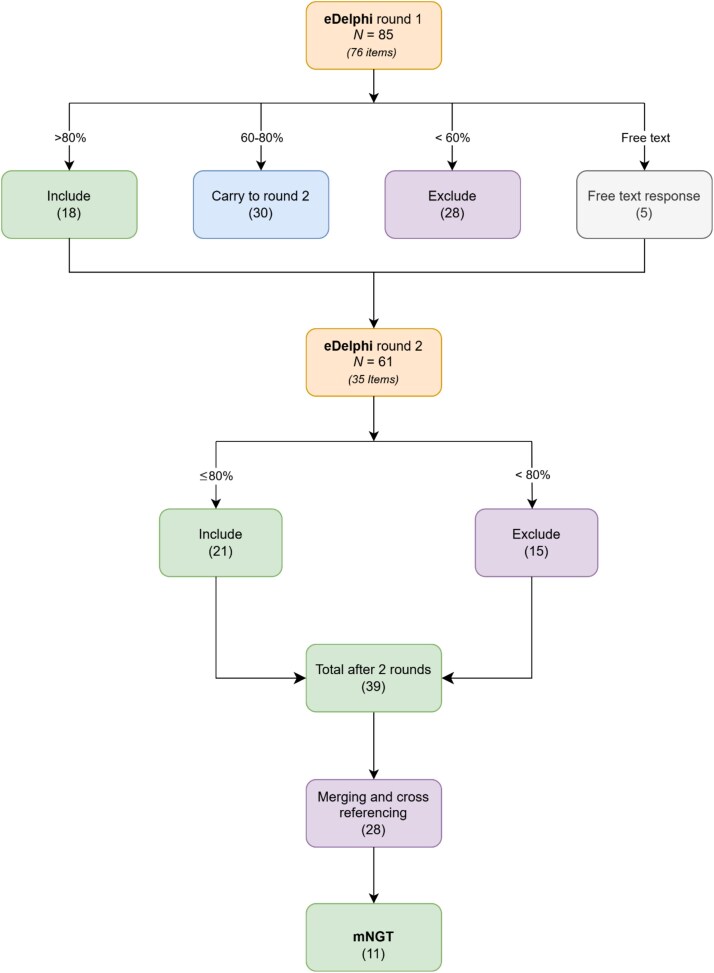
The process of selecting items for inclusion in the referral template using eDelphi and m-NGT.

Out of 76 items initially presented, 18 items were scored as critically important in Round 1 and were retained. Twenty-eight items were excluded for scoring <60%. Examples of items that did not reach consensus included whether depression screening had been completed and details of recent hospitalisations or frailty. The remaining 30 items, which scored between 60% and 80%, were carried forward to Round 2 for re-evaluation (see Figure 1). Five additional items were added to Round 2, having been suggested by two or more panel members through the free-text option in Round 1. These new items were related to driving, disclosure of a diagnosis and enduring power of attorney.

In Round 2, 21 items surpassed the 80% consensus threshold and were considered critically important. Therefore, in total, 39 items were retained after both rounds of the e-Delphi study (See Figure 1). Items scoring most critical in Round 1 included those related to presenting symptoms, collateral history, safeguarding and medications. In Round 2, items pertaining to initial workup and behavioural or functional changes scored over the >80% threshold. A detailed list of all critically important items from both rounds is provided in Table 2.

**Table 2:** Items considered critically important in round 1 & round 2

Items deemed critically important^*^ (≥80%) (Round 1, n = 85, Round 2, n = 61) ^*^Critically important = score of 8–9 on Likert scale	Round 1 (%)	Round 2 (%)
**Patient details**		
Age	84	
Known disabilities (e.g. Intellectual Disability/Down syndrome)		82
Primary language		85
Interpreter required	87	
Able to attend clinic	80	
Caregiver details/relationship to the patient	86	
Urgency of referral		83
**Contact details**		
Patient	95	
Caregiver	93	
Referring doctor	87	
Usual GP	84	
**Presenting symptoms/complaint**		
The specific reason for referral	94	
Presenting symptoms of concern, e.g. short-term memory loss, word finding difficulties	88	
Has there been rapid deterioration?	81	
Is there a pre-existing diagnosis of dementia?	88	
Observed decline reported by patient and/or caregiver	87	
**Collateral history**		
Collateral history findings	82	
**Safety/safeguarding**		
Patient safety concerns (e.g. falls, getting lost in public places)	91	
Safeguarding concerns (e.g. physical abuse, psychological abuse, neglect)	89	
Safety concerns for carers	87	
**Past medical history**		
Psychiatric history		88
Known deafness		80
**Medications**		
Current medications	99	
**Social/family history**		
Living arrangements (e.g. Living alone)	82	
Alcohol and substance use history		82
**Cognitive screening findings**		
Cognitive screening completed (yes/no)		87
Cognitive screening scores		83
**Investigations findings**		
Neuroimaging findings (CT Brain or MRI Brain)		82
**Functional changes**		
Problems with self-care (bathing, dressing, cleaning)		87
Problems with daily activities such as cooking or shopping		87
How long have memory problems been present?		87
**Behaviour changes**		
Agitation		87
Aggression		87
**Other**		
Patient-specific concerns		90
Caregiver-specific concerns		87
**Additional statements Round 2**		
Is the patient aware of the reason for the referral?		82
Has a dementia diagnosis been disclosed?		87

### Qualitative feedback from the e-Delphi panel

In addition to the quantitative scores, participants provided valuable qualitative comments that highlighted key considerations for the referral template. This feedback highlighted the critical tension between the need for comprehensive clinical information and the practical realities of clinician workload, including time constraints and the avoidance of duplicated effort. Several GP participants expressed concern regarding feasibility and acceptability, stressing the need for consideration of practical application. For example, one participant remarked, ‘Clinical information is useful, but forms should not be too long, and work should not be duplicated if possible’. Another expressed concern regarding the time constraints of GPs, noting that ‘a long and non-practical referral form could act as a significant barrier, especially where GPs are stretched for time’.

Secondary care clinicians also expressed some concerns in their comments, primarily related to duplication of work. For example, one participant requested that the referring GP ‘avoid duplication of referral’ while others noted that ‘much of the info will need to be re-assessed *de novo* in the appointment’. This indicates the need to balance comprehensive information with the practicalities of a clinician’s workflow, directly informing the subsequent m-NGT process.

### Modified nominal group technique

Following the e-Delphi rounds, the 39 remaining items were cross-referenced with a generic referral template and merged where appropriate, resulting in a list of 28 items for consideration during the m-NGT session. The PPI panel reviewed these items, with a particular focus on those related to patient or caregiver safety or concerns. They provided informed feedback on several items that they considered non-critical in the referral stages, such as caregiver contact details, relationship to the patient, safeguarding concerns and safety concerns for caregivers. The feedback from the PPI panel was carried forward to the m-NGT process for further consideration and inclusion in the preparatory task.

**Figure 2:**
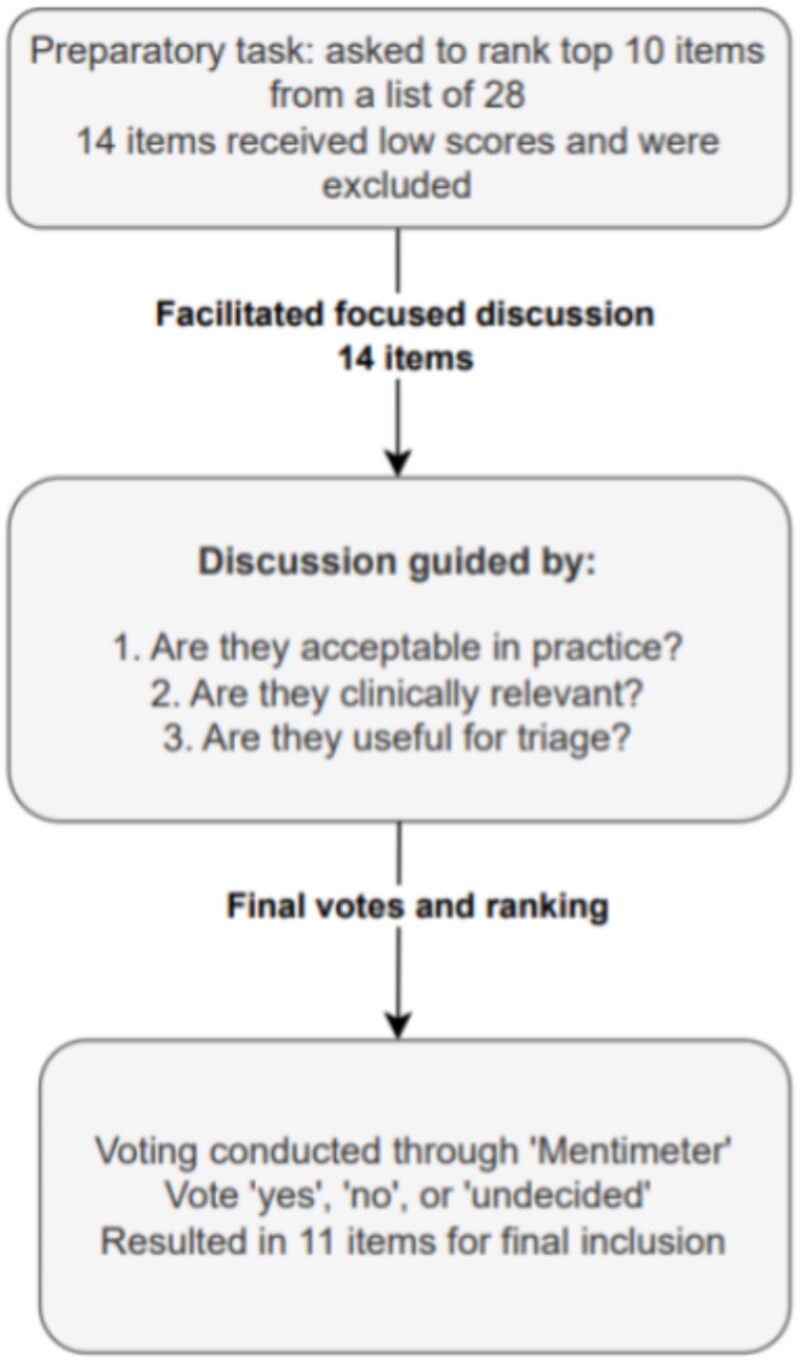
Flow diagram of the m-NGT process.

**Figure 3:**
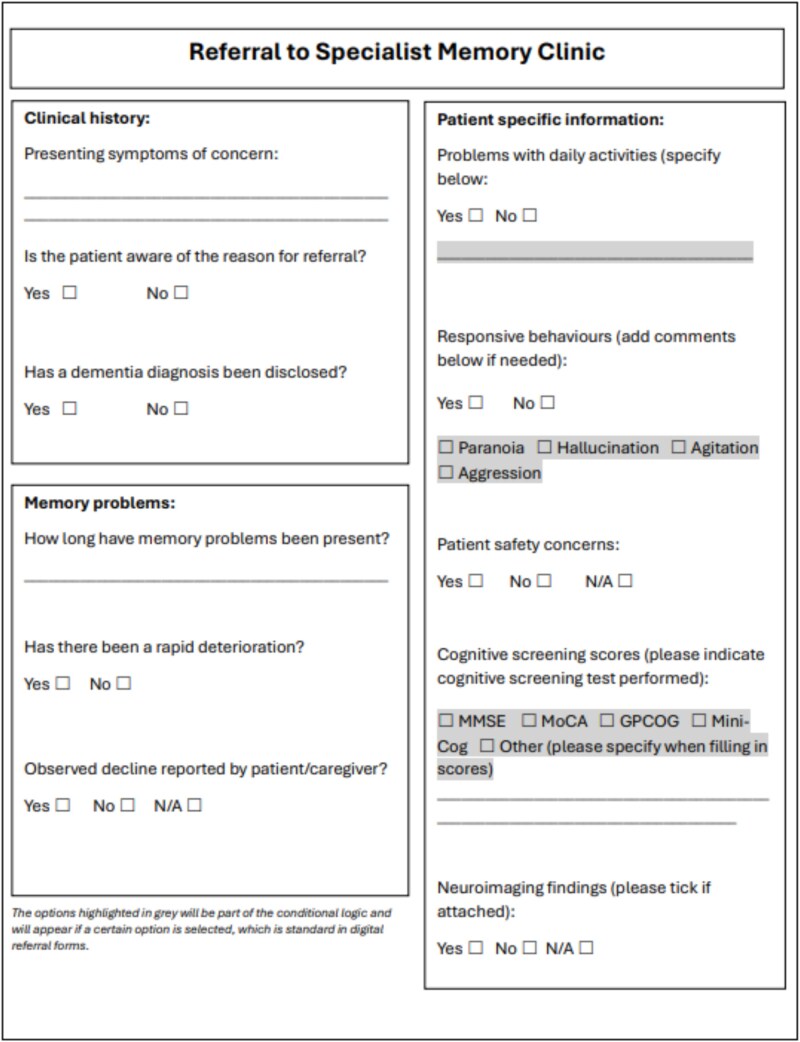
Referral template co-designed for use in an electronic format.

The preparatory task, where the research team were asked in advance of the meeting to rank the top ten from the remaining 28, resulted in the number of items being reduced to 14. An 80-minute online meeting was held to co-design the referral template using the modified m-NGT, with participation from 10 members of the research team (five GPs, one geriatrician, one neurologist, one psychiatrist and two nurses). The Delphi results, cross-referencing, and PPI contributions, along with the results of the preparatory task, were presented at the outset of the meeting. Following this, the attendees debated the items for inclusion, followed by the m-NGT voting; 11 items were chosen for inclusion in the referral form (See Figure 2). Notably, two items that were ultimately included in the final referral template were first proposed by participants during the first round of the survey. A complete record of items, including those retained and those excluded at each stage, is provided in Appendix 1 in the supplementary data section. The final referral template co-designed for use in an electronic format is presented in Figure 3.

## Discussion

This study has addressed a critical gap in dementia care referral pathways by developing a consensus-based, bespoke referral template for suspected dementia, designed to improve communication between GPs and secondary care memory services. Through a robust, sequential mixed-methods approach combining an e-Delphi survey with an m-NGT, the study successfully identified and prioritised the essential components of a referral template. The process systematically distilled an initial list of 76 potential referral template items into a final set of 11 items, thereby transforming a broad selection of useful information into a concise list of essential items for effective referral and triage, balancing clinical relevance with template usability.

An analysis of the items that achieved the highest levels of consensus reveals a clear clinical logic. Items prioritised, such as ‘cognitive screening scores’, ‘rapid deterioration,’ ‘problems with daily activities’ and ‘patient safety concerns’, underline their importance in dementia diagnosis. These domains reflect established practice guidelines, which emphasise cognitive testing and collateral history as cornerstones of dementia assessment [20, 21]. While the risk of duplication of testing was considered, cognitive screening scores were retained by the panel as a critical component for triage; this inclusion is intended to support, rather than replace, the detailed qualitative and quantitative assessments undertaken by secondary care specialists. Furthermore, these findings align with existing evidence demonstrating that GP referral letters containing specific diagnostic criteria, including ‘interference with daily functioning’ and ‘objective cognitive decline’, have a significantly higher predictive value for a subsequent dementia diagnosis at a memory clinic [22]. The consensus process employed in this study has, therefore, effectively identified these key predictive data points, incorporating them into a structured format. The inclusion of ‘patient safety concerns’ is particularly notable. Although omitted from most of the referral templates reviewed as part of the survey development for this study, this was considered essential for triage and risk assessment. This aligns with recent calls for dementia pathways to better integrate safeguarding and caregiver wellbeing into diagnostic processes [8]. By providing a clear framework, the template guides GPs to compile patient data into a predictive summary, thereby improving the diagnostic pathway’s quality and efficiency.

The items excluded from the final template are equally informative. Items such as ‘depression screening completed,’ ‘details of recent hospitalisations,’ or ‘frailty’ did not reach the pre-determined consensus threshold. This does not diminish their clinical importance. Rather, their exclusion highlights the specific purpose of this template as a referral tool, rather than as a comprehensive diagnostic guide. Effective referral communication relies on conveying a core dataset of essential information necessary for optimal patient triage [23]. Accordingly, the expert panel prioritised information deemed essential for the referral (e.g. presenting symptoms), risk assessment and triage (e.g. safety concerns), and objective point-in-time measures (e.g. cognitive screening scores). This suggests the panel prioritised the core items needed to triage the patient over other information that, while part of a standard workup, was less essential for the referral itself.

Qualitative feedback highlighted the need for template brevity and the avoidance of duplication, reflecting the demanding workload in primary care. This reflects a well-documented challenge in healthcare, where fragmented referral systems and poor communication can impede care coordination, whereas concise, structured referral templates can streamline care pathways [24]. Our findings echo previous research findings that found that effective templates must balance process efficiency with patient-centredness [25]. Against this backdrop, the m-NGT session functioned as more than a final selection exercise; it was a critical forum for negotiation. By structuring the discussion around clinical relevance, practical acceptability and triage value, the group balanced these competing needs. The resulting template is a practical compromise; a tool that is clinically robust while remaining pragmatically feasible for its end-users. The template therefore aims to support GP decision-making, ensuring a consistent and high-quality referral process without creating a prescriptive tool that might limit professional autonomy.

The importance of this refined referral process is further amplified by the emergence of anti-amyloid disease-modifying therapies for Alzheimer’s disease. These novel treatments are most effective in the early stages of the disease and are accompanied by strict eligibility criteria. This creates an urgent need for diagnostic pathways that can efficiently and accurately identify potentially eligible candidates from their first presentation with cognitive concerns in primary care. Our co-designed template directly supports this need by structuring the referral around the core items essential for this initial triage, such as cognitive screening scores, observed functional decline and the timeline of deterioration. By standardising this crucial first step, the template acts as an enabling tool, helping to fast-track appropriate patients towards the comprehensive assessments required for DMT consideration.

### Strengths and limitations

A primary strength of the study is the robust, phased design, which combined a broad e-Delphi process with an m-NGT for structured deliberation. The inclusion of a diverse, multidisciplinary m-NGT clinical panel and the active involvement of PPI participants ensured the final template is balanced and relevant. Our approach demonstrates the value of embedding caregiver voices in pathway design to ensure that referral processes are person-centred and not solely clinician-driven.

However, limitations must be acknowledged. Our findings are specific to the Irish healthcare system, potentially limiting the template’s direct transferability without local adaptation. While the identified core items likely have international relevance, their direct applicability may vary across systems with different referral structures. Recruitment via professional networks may have introduced selection bias. Crucially, the template is a consensus-based instrument that has not yet undergone prospective validation in a clinical setting. Its real-world feasibility, usability, and potential for digital integration into existing electronic health records remain theoretical, making a pilot study to evaluate its implementation the essential next step.

## Conclusion

This study successfully addressed a critical gap in the dementia diagnostic pathway by developing an evidence-informed referral template through a rigorous, multi-stage consensus-building process, addressing variability and inefficiency in current referral practices. Essential items have been identified and prioritised, creating a practical tool that balances the need for comprehensive clinical information with the realities of primary care workloads. The resulting co-designed template serves to standardise communication and improve the quality of information at the primary-to-secondary care transition. By doing so, the referral template holds the potential to streamline clinical pathways, reduce triage delays and support a more timely and accurate diagnosis for individuals with suspected dementia. Ultimately, this co-designed tool is more than a template; it is a crucial step towards creating a more responsive, efficient and person-centred dementia diagnostic pathway, ensuring individuals receive the right care at the right time in an evolving therapeutic landscape.

## Supplementary Material

aa-25-2907-File002_afag008

## Research Data Transparency and Availability:

The data underlying this article will be shared on reasonable request to the corresponding author.
