# Supplementary material for: Co-designing a structured referral template to enhance dementia diagnosis: a modified e-Delphi study
Source: Age Ageing. 2026 Feb 2;55(2):afag008. doi: 10.1093/ageing/afag008 (PMC12862972; doi:10.1093/ageing/afag008)
Supplement: aa-25-2907-File002_afag008 [file aa-25-2907-file002_afag008.pdf]

Co-designing a Structured Referral Template to Enhance Dementia Diagnosis: A modified e-Delphi study. Supplementary material: Appendix 1

Items for inclusion	Round 1 Delphi	Round 2 Delphi	Preparatory ranking task	m-NGT
Gender	Exclude			
Age	☑		Exclude - cross referenced with generic referral form	
Marital status	Exclude			
Ethnic origin	Exclude			
Education level	Exclude			
Known disability (Intellectual disability/Down syndrome)	Carry to round 2	☑	☑	Exclude
Primary language	Carry to round 2	☑	☑	Exclude
Interpreter required	☑		☑	Exclude
Able to attend clinic	☑		☑	Exclude
Caregiver details/relationship to patient	☑		PPI recommended to exclude	Exclude
Preference for virtual review/phone/domicilary review	Exclude			
Urgency of referral	Carry to round 2	☑	Exclude - cross referenced with generic referral form	
Contact details of patient	☑		Exclude - cross referenced with generic referral form	
Contact details of caregiver	☑		PPI recommended to exclude	Exclude
Contact details of referring doctor	☑		Exclude - cross referenced with generic referral form	
Usual GP	☑		☑	Exclude
Details of nominated healthcare representative	Carry to round 2	Exclude		
Specific reason for referral	☑		Exclude - cross referenced with generic referral form	
Presenting symptoms of concern e.g. short term memory loss, word finding difficulties	☑		☑	☑
Have differential diagnoses/other causes been excluded for these symptoms e.g. delirium	Carry to round 2	Exclude		
Formal screening for depression	Exclude			
Positive findings on neurological examination	Exclude			
Has there been a rapid deterioration?	☑		☑	☑
Is there a pre-existing diagnosis of dementia?	☑		☑	Exclude
Any recent hospitalisations or acute illnesses?	☑			
Observed decline reported by patient and/or caregiver?	☑		☑	☑
Has collateral history been obtained?	Carry to round 2	Exclude		
Collateral history findings	Carry to round 2	☑	☑	Exclude
Risk of imminent hospital admission	Exclude			
Risk of emergency residential care admission	Carry to round 2	Exclude		
Patient safety concerns (e.g. falls, getting lost in public places)	☑		☑	☑
Safeguarding concerns (e.g. physcial abuse, psychological abuse, neglect)	☑		PPI recommended to exclude	Exclude
Safety concerns for carers	☑		PPI recommended to exclude	Exclude
Cardiovascular history	Carry to round 2	Exclude		
Psychiatric history	☑	☑	☑	Exclude
Frailty	☑			
History of delirium	Carry to round 2	Exclude		
Known deafness	☑	☑	☑	Exclude
Has had a hearing test	Exclude			
Visual impairment	Exclude			
Has had an eye sight test	Exclude			
Current medications	☑		Exclude - cross referenced with generic referral form	
PRNs (as required medications)	Carry to round 2	Exclude		
Allergies	Carry to round 2	Exclude		
Adverse drug events	Carry to round 2	Exclude		
Living arrangements (e.g. living alone)	☑		☑	Exclude
Type of accommodation	Exclude			
Alcohol and substance use history	☑	☑	☑	Exclude
Smoker	Exclude			
Occupation	Exclude			
Family history of dementia	Exclude			
Cognitive screening completed (yes/no)	☑	☑	**	☑
Cognitive screening scores	☑	☑	**	☑
Generalised Anxiety Disorder score (GAD)	Exclude			
Recent blood test results	Carry to round 2	Exclude		
Neuroimaging findings (CT Brain or MRI Brain)	☑	☑	☑	☑
Chest x-ray result	Exclude			
Electrocardiagram (ECG) result	Exclude			
Problems with self-care (bathing, dressing, cleaning)	☑	Exclude		
Problems with daily activities such as cooking or shopping	☑	☑	☑	☑
How long have memory problems been present?	☑	☑	☑	☑
Anxiety	Exclude			
Depression	Exclude			
Sleep disturbances	Exclude			
Paranoia	☑	☑	*	☑
Hallucinations	☑	☑	*	☑

Agitation	☑	☑	*	☑
Aggression	☑	☑	*	☑
Community service invovlement "Alzheimer Society of Ireland/Dementia Advisor	Exclude			
Does the patient have support from a member of the Primary Care Team (e.g., Physio, Occupational Therapy, Public Health Nurse, Psychologist, etc.)?	Exclude			
Does the patient have support from the Integrated Care of Older Persons service?	Exclude			
Has the patient previously been referred to specialist services such as neurology, gerontology, psychiatry, Allied Healthcare professionals.	Exclude			
Home help supports	Exclude			
Patient specific concerns	☑	☑	Exclude	
Caregiver specific concerns	☑	☑	Exclude	
Disucssion of future care planning	Exclude			
Is the patient aware of the reason for referral? (came from free text)		☑	☑	☑
Has a dementia diagnosis been disclosed? (came from free text)		☑	☑	☑
* = combined for preparatory ranking task into "responsive behaviours"				
** = combined for preparatory ranking task into "cognitive screening scores"				
